# HypoAware-a brief and partly web-based psycho-educational group intervention for adults with type 1 and insulin-treated type 2 diabetes and problematic hypoglycaemia: design of a cost-effectiveness randomised controlled trial

**DOI:** 10.1186/s12902-015-0035-0

**Published:** 2015-08-21

**Authors:** Stefanie MPA Rondags, Maartje de Wit, Maurits W van Tulder, Michaela Diamant, Frank J. Snoek

**Affiliations:** Department of Medical Psychology, VU University Medical Centre, Van der Boechorststraat 7 (D-342), Amsterdam, 1081 BT The Netherlands; EMGO+ Institute for Health and Care Research, VU University Medical Centre, Amsterdam, The Netherlands; Department of Epidemiology and Biostatistics, VU University Medical Centre, Amsterdam, The Netherlands; Department of Internal Medicine & Diabetes Center, VU University Medical Centre, Amsterdam, The Netherlands; Department of Medical Psychology, Academic Medical Center (AMC), Amsterdam, The Netherlands

## Abstract

**Background:**

Problematic hypoglycaemia is a significant problem among people with insulin-treated type 1 and 2 diabetes mellitus, which adversely affects quality of life and leads to high societal costs. Blood glucose awareness training (BGAT) is a psycho-educational group intervention that has shown to be effective but difficult to implement in clinical practice, due to its demanding nature. The aim of this study is to evaluate the cost-effectiveness of the HypoAware intervention that has its roots in BGAT and helps patients to effectively recognize, treat and prevent hypoglycaemia, while also focussing on the psychosocial consequences of living with the constant risk of hypoglycaemia, both for patients and their significant others.

**Methods/design:**

An economic evaluation will be conducted alongside a cluster-randomised controlled trial in eight hospitals in the Netherlands. We aim to include 154 participants who will either receive HypoAware or care as usual. HypoAware consists of three group sessions and is combined with two online modules. The primary clinical outcome is severe hypoglycaemia. Secondary outcomes include mild hypoglycaemia, hypoglycaemia awareness, fear of hypoglycaemia, diabetes distress, anxiety and depression, health-related quality of life, diabetes-specific self-efficacy and HbA1c. Utilities will be measured using the EQ-5D-5 L questionnaire. Costs will be measured from a societal perspective and include health care utilization, medication and lost productivity costs. Measurements will be performed at baseline, 2, 4, and 6 months to compare both groups, and an additional 12 months for the intervention group only. Process outcomes will be gathered after every group meeting via telephone/email contact with health care professionals and via the online environment.

**Discussion:**

HypoAware is expected to significantly reduce episodes of severe hypoglycaemia, with subsequent beneficial effects on psychosocial outcomes and health care costs. When proven cost-effective, HypoAware will be incorporated in the clinical guidelines of Dutch diabetes care.

**Trial registration:**

Dutch Trial Register NTR4538

## Background

Hypoglycaemia is the most common adverse event associated with insulin treatment in type 1 (T1DM) and type 2 diabetes (T2DM) and causes morbidity, a significant reduction in quality of life, and even death [1–3]. Hypoglycaemia can lead to high costs due to increased health care consumption and work productivity loss [1, 4]. Psycho-educational interventions, like Blood Glucose Awareness Training (BGAT), aimed at reducing and preventing hypoglycaemia are effective [5], but are demanding on clinics’ resources, health care professionals and people with diabetes. Current economic climate demands a cost-effective approach. Therefore, we developed a brief group intervention based on key ingredients of BGAT, called HypoAware. We combined three group sessions of 2.5 h each with an online learning environment, to maintain quality and improve attractiveness, while keeping costs at a minimum.

We have previously conducted a multi-centre pilot study of HypoAware in people with T1DM and T2DM and problematic hypoglycaemia doi: 10.1111/dme.12876. This study demonstrated the intervention’s feasibility and acceptability. Continuing on this pilot study, we designed a two-arm cluster-randomised controlled trial comparing HypoAware with care as usual. In addition to health-related effectiveness, cost-effectiveness will also be assessed, since this will inform policy and decision makers in diabetes care about the cost/benefit ratio of HypoAware.

This study aims to evaluate the effectiveness and cost-effectiveness of a brief blended psycho-educational intervention called HypoAware, aimed at reducing hypoglycaemia-related problems in people with T1DM and T2DM in comparison with usual care.

## Methods/design

### Design

An economic evaluation from a societal perspective will be performed alongside a two-arm multi-centre, cluster-randomised controlled trial with follow up measurements at 2, 4 and 6 months for both groups. Additionally a 12 months follow-up measurement will take place in the intervention group only to analyse long-term effects over time.

### Ethical approval

The study protocol was approved by the Ethics Committee of the VU University Medical Centre certified by the Central Committee on Research involving Human Subjects in the Netherlands (NL47354.029.13, registration number 2014.007). We will obtain written informed consent from all participants.

### Setting

The study will be carried out in eight self-selected outpatient diabetes clinics in the Netherlands. The diabetes care teams in these clinics will conduct the recruitment and intervention phase. All teams have appointed a local coordinator. The study coordinator (SR) will be in close contact with the local coordinators throughout the study.

### Randomisation

Cluster randomisation will be done prior to recruitment of participants at the level of the participating clinics to avoid contamination within the clinics. The participants in the control group receive the intervention after the 6 months follow-up as an incentive to participate. Random allocation will be performed by two members of the study team, who randomly select notes from two opaque envelopes, one with the eight names of the clinics and one with four notes with ‘intervention’ and four notes with ‘control’. Participants will be allocated based on the clinic where they are registered.

### Participants

Persons are eligible for this study if they are 18 years or older, are treated for T1DM or T2DM in an outpatient setting, perform at least three multiple daily insulin injections a day or are on continuous subcutaneous insulin infusion (pump therapy) and have experienced one or more episodes of SH in the past two years and/or have subjective impaired awareness of hypoglycaemia (the patient has no or significantly reduced autonomic symptoms when blood glucose drops below 4 mmol/l). Furthermore, patients should have access to the Internet and be willing and able to actively attend the three group meetings.

Patients are excluded from participation when they meet one or more of the following exclusion criteria: serious medical co-morbidity (e.g. cancer or dialysis); a major psychiatric disorder (e.g. bipolar depression or schizophrenia); established cognitive impairment; substance abuse; pregnancy; insufficient Dutch language skills or visual impairments.

### Recruitment and planning

**Fig. 1:**
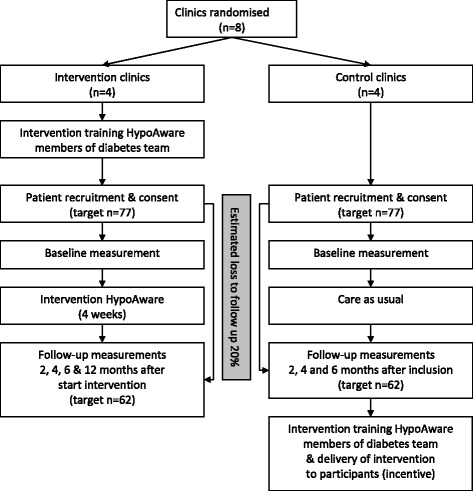
Flow chart of study design and participant flow

In order to take part in our study, clinics need to indicate to be able to include 20 participants. Via posters and information brochures, the study will be announced and explained. The diabetes teams in each clinic will check patients on in—and exclusion criteria. The local coordinator will collect all informed consent forms and send those to the study coordinator. Patients will receive a personal link to the online questionnaires via email. Then, participants are either enrolled in HypoAware or will first receive usual care for 6 months, depending on the clinic at which they are registered. Blinding of participants is not possible due to the nature of the intervention. Participants receive the online questionnaire again at 2, 4 and 6 months follow-up. After 6 months, our study team will train the diabetes care teams from the control group. Participants from the control group can receive the intervention if so wished. Only the intervention group receives an additional questionnaire after 12 months. An overview of the study design and participant flow is provided in Fig. 1.

### Interventions

#### HypoAware

As mentioned earlier, HypoAware is an adapted version of BGAT. HypoAware was developed in close collaboration with diabetes experts and persons with diabetes. HypoAware consists of three group sessions of 2.5 h during four weeks, combined with two online modules in the weeks between meetings. Topics covered in the group meetings and online are presented in Table 1. Groups are led by two trained diabetes professionals and consist of eight participants; due to the nature of the intervention these professionals cannot be blinded. HypoAware aims to improve symptom recognition, risk awareness, preventive and problem-solving strategies and coping with (the risk of) hypoglycaemia. The intervention is guided by the biopsychobehavioural model of risk of severe hypoglycaemia by Linda Gonder-Frederick and colleagues [6], which describes the effect of behavioural mechanisms, like attention for and attribution of hypoglycaemia symptoms, on the risk of severe hypoglycaemia. It also focuses on the usage of ‘internal cues’ for low blood glucose (physical and mental symptoms) and the prediction and prevention of hypoglycaemia through ‘external cues’ (e.g. the effect of insulin, food intake and physical activity). Group sessions are highly interactive and aimed at patient-empowerment, in which patient responsibility and informed decision making is encouraged (when appropriate). Group meetings promote modelling, social reinforcement and sharing of experiences. Partners or other significant others are invited to the third meeting to discuss their attitudes towards their own and partners’ responsibilities regarding the prevention and treatment of hypoglycaemia. The user-friendly online modules consist of educational texts and videos, automated exercises, self-tests and personal diary analyses. Results and progress can be viewed online and printed by participants and trainers and are discussed in the group meetings. Also, results can be entered in the participants’ medical file for subsequent routine visits. More information about the development and feasibility of HypoAware is published elsewhere doi: 10.1111/dme.12876.

**Table 1:** HypoAware: development and pilot-study of a brief and partly web-based psychoeducational group intervention for adults with type 1 and insulin-treated type 2 diabetes and problematic hypoglycaemia

Group meetings	Online environment
1b) First group meeting: introduction, discussion of goals, ‘internal cues’ of hypoglycaemia and impaired hypoglycaemia awareness, ‘feel-exercise’ to scan for internal cues, psychobehavioural mechanisms in the treatment of hypoglycaemia, explanation of homework—the diary, estimation-grid an internal cues. (2.5 h)	1a) Self-test for goal-setting
	1c) Homework: two weeks of keeping a blood glucose estimation and symptom diary, online education synchronous to topics of first group meeting plus tips to prevent hypo- and hyperglycaemia and playful tests to scan for neuroglycopenic symptoms. (1 h)
2a) Second group meeting: discussion of first online diary analysis, influence of ‘external cues’ on blood glucose level, fear of hypoglycaemia—degree of worrying about hypoglycaemia and taking precautionary measures versus risk of hypoglycaemia, explanation of homework—external cues. (2.5 h)	2b) Homework: two weeks of keeping a blood glucose estimation and symptom diary, online education synchronous to topics of second and third group meeting plus tips to treat hypoglycaemia, education about counting carbohydrates, the working of insulin and the effect of exercise, stress and illness on blood glucose, playful ‘experiments’ with food, exercise and insulin, a stress reduction exercise and a ‘hypo-simulator’ exercise which show effect of different foods on blood glucose. (1,5 h)
3a) Third group meeting (significant others are invited): introduction of partners, role of partner versus needs of participant concerning the risk of hypoglycaemia, discussion of second online diary analysis, discussion of accomplished goals, setting of specific goals for the action plan, which will be followed-up on in the next diabetes consultation. (2.5 h)	3b) Self-test for goal-accomplishment and future goal-setting and action plan^a^
	End-document with intervention results (self-tests, two diary analyses, experiment results and action plan) can be printed or digitally saved by the trainers and participants. Results, remarks and referrals will be entered in the participants’ medical file.

Table from doi: 10.1111/dme.12876 in adapted form

#### Training

Prior to delivering HypoAware, members of the diabetes teams from the intervention group (two to four members per clinic) receive a one-day protocolized training by our study team and are provided with a detailed trainer manual. Eligible for the training are diabetes nurses, endocrinologists, diabetes dieticians or medical psychologists with expertise in diabetes. The training focuses on how to coordinate and implement the program and how to lead the group meetings. The HypoAware approach encourages future trainers to stimulate participants in a non-judgmental and non-directive way to share their experiences and learn from each other rather than from a tutor. The focus is on informed decision-making and patient-empowerment.

During the study, trainers are contacted after each session, to check for any protocol violations and experienced difficulties. Trainers can contact the study coordinator to discuss any questions or problems should they arise.

#### Control-care as usual

In the first six months, participants of the control group only have access to care as normally provided by their diabetes team. There is no specific protocol or guideline available to guide care for people with diabetes and problematic hypoglycaemia other than the national guidelines [7]. According to survey data by our research group (unpublished), patients can expect to receive one to three extra consultations with the diabetes nurse and/or dietician and additional telephone/email contact. The appointments with the diabetes nurse focus on adjusting glucose-lowering medication and examining blood glucose patterns. Often (temporary) higher blood glucose targets are advised. The dietician helps with adjusting the carbohydrate-insulin ratio.

After the six months follow-up, participants in the control group can receive HypoAware.

### Outcomes

All measures mentioned below will be administered at the start of the study, and 2, 4 and 6 months after inclusion through web-based questionnaires. The clinical data will be collected from the medical files by the local coordinator from each clinic. The intervention group receives an additional measurement at 12 months to analyse long-term effects over time.

#### Primary outcome

The primary outcome is *self-reported frequency of severe hypoglycaemia* after 6 months, defined as “a hypoglycaemic event serious enough to require the help of another person”.

#### Secondary health-related outcomes

- *Self-reported frequency of mild hypoglycaemia* per week is formulated as “a hypoglycaemic event not serious enough to require the help of another person”. We will ask participants to count the number of blood glucose measurements in the past week, how many of those measurements were below 4 mmol/l and how many times they felt low without confirming it with a test.
- *Hypoglycaemia awareness* will be evaluated using the validated Gold score [8] and Clarke questionnaire [9].
- *Fear of hypoglycaemia*(both worrying and avoidance behaviour) will be measured using the revised Hypoglycaemia Fear Survey (HFS-II) [10].
- *Diabetes-related distress* will be assessed with the short-form Problem Areas In Diabetes scale (PAID-5) [11].
- *Anxiety and depression*, will be assessed with the Hospital Anxiety and Depression Scale (HADS) [12].
- *Diabetes-specific self-efficacy* will be evaluated with the Confidence In Diabetes Self-care (CIDS) [13].
- *Health-related quality of life* will be measured using the EuroQol 5-dimensional 5-level version (EQ-5D-5 L) and the visual analogue scale (EQ-5D VAS) [14].
- Demographics will be measured at baseline, along with the following clinical data from the medical file: glycosylated haemoglobin (HbA1c), type of diabetes, treatment regimen, diabetes duration, diabetes-related complications, and co-morbidities.

#### Cost-effectiveness outcomes

The obtained EQ-5D-5 L scores [14] will be used to calculate utilities according to the Dutch tariff. For the economic evaluation, costs will be measured using the TiC-P questionnaire [15] with a recall period of 2 months. Costs that will be included are direct costs of healthcare utilization (e.g., hospital admissions, outpatient visits and calls, emergency room visits, ambulance transfers, medication and medical supply usage), costs of informal care, and indirect costs (productivity loss due to work absenteeism from paid and unpaid work). If available, Dutch guideline prices will be used to value resource use. Medication use will be valued using prices of the Royal Dutch Society for Pharmacy. Lost productivity costs will be calculated according to the friction cost approach (friction period 154 days) using the mean age and sex-specific income of the Dutch population. According to the friction cost approach a sick employee is replaced after a certain amount of time (the friction period) after which there are no lost productivity costs anymore. All costs will be adjusted to the year in which most data is collected using consumer price indices.

#### Process outcomes

We will assess participants’ and trainers’ compliance with the intervention via contact with the trainers after every group meeting. We will determine the number of meetings followed by every participant and protocol violations. We will also use information from the online environment to determine whether participants completed the online assignments.

### Sample size

A power analysis was performed to determine the sample size using STATA 11.2 (Statacorp, Texas, USA). We based our effect size on a randomised controlled trial of the intervention BGAT that showed the most conservative effect [16]. Mean frequency of severe hypoglycaemia at baseline was 1.61 (SD 3.49) and 0.13 (SD 0.33) 6 months post BGAT. The control condition (group education about diabetes guided by a physician) scored 1.76 (SD 3.71) at baseline and 1.07 (SD 2.85) after 6 months. This implicated that, with 3 follow-up measurements and ρ = 0.6, the alpha set at 0.05, a power of 80 %, and adjusting for clustering within 8 diabetes teams with an ICC of 0.01 as described by Twisk [17], we need 62 patients per group (total *n* = 124) to perform our primary analyses. Anticipating a 20 % loss to follow-up, we aim to randomise 77 patients per group (total *n* = 154).

### Statistical analyses

Data will be analyzed based on the intention-to-treat principle. We will use a multilevel generalized linear model for count data to assess the effectiveness of HypoAware on the frequency of severe hypoglycaemia, while allowing for clustering of events in patients and clinics. Secondary outcomes will also be assessed using multilevel (generalized) linear models in case of continuous, count or ordinal outcome variables. Generalized Estimation Equations (GEE) will be used to analyse dichotomous outcome variables.

#### Economic evaluation

Both a cost-effectiveness and cost-utility analysis will be performed with a time horizon of 6 months. Therefore, discounting is not necessary.

Societal costs will be related to the following effect measures in the economic evaluation: 1) Frequency of severe hypoglycaemia; 2) Quality-adjusted life-years (QALYs), calculated using the area-under-the-curve method with linear interpolation between time points. QALYs will be based on the Dutch tariff for the EQ-5D-5 L.

The analysis will be done according to the intention-to-treat principle. Missing cost and effect data will be imputed using multiple imputation techniques. Adjustment for confounders and effect modifiers will be done, if necessary. Sensitivity analyses will be performed to assess the robustness of the results using different assumptions regarding costs.

Incremental cost-effectiveness ratios (ICERs) will be calculated by dividing the difference in mean total costs between the HypoAware and usual care groups by the difference in mean effects. Bootstrapping with 5000 replications will be used to estimate 95 % confidence intervals around cost differences and the uncertainty surrounding the ICERs. Uncertainty will be graphically presented on cost-effectiveness planes. Cost-effectiveness acceptability curves showing the probability that the intervention is cost-effective in comparison with usual care for a range of different ceiling ratios will also be estimated.

## Discussion

Existing psycho-educational interventions aimed at reducing hypoglycaemia are effective, but demanding on the recourses of the clinics, health professionals and people with diabetes. We propose to study the costs and effects of our brief psycho-educational intervention, HypoAware, in comparison to care as usual in a cluster-randomised RCT.

A potential limitation of this study could be our relatively short follow-up period of 6 months to compare group effects. It is possible that the potential benefits of HypoAware will not yet be fully visible after 6 months, because severe hypoglycaemia occurs on average 1.0 to 1.7 times a year [18]. However, in this specific group with impaired awareness of hypoglycaemia and/or recent severe hypoglycaemia, occurrence of severe hypoglycaemia is higher than in a non-selected group of diabetes patients. To study the longer term effects, we do measure the intervention group at an additional 12 months. It would not be ethical to withhold the participants in the control group our new intervention for a year, since no other structured care is available for these patients.

Another possible limitation is that we rely on self-report of hypoglycaemia since validation of hypoglycaemic episodes, for example by means of continuous glucose monitoring technology, was not feasible. Self-reported frequency of severe hypoglycaemia for the past year (and the past week for mild hypoglycaemia) seems reliable, although an underestimation of severe hypoglycaemia seems likely in case of sub-groups with a high frequency of severe hypoglycaemia [19]. In our study, we will inquire about the previous six months in case of severe hypoglycaemia.

It is not possible to blind patients and caregivers to the randomisation due to the nature of the study; however, since both groups receive HypoAware, we can assume similar expectancies in both groups.

Overall, this study will provide valuable information about the effectiveness and cost-effectiveness of a combined group and online psycho-educational intervention to reduce and prevent episodes of severe hypoglycaemia and other hypoglycaemia-related problems in type 1 and type 2 insulin-treated diabetes patients, both from a clinical and societal perspective. Since, in the current economic climate, care has to be as effective as possible, for as little money as possible, interventions have a greater chance of being implemented if cost/benefit trade-off is favourable. Studying our intervention will also contribute to the evidence of the effect of downsizing relatively ‘intensive’ and costly interventions and complementing a group format with the Internet. This format could be applied to other interventions to improve implementation, while maintaining quality and high standards of care.

If HypoAware is proven cost-effective, it will be adopted and promoted by the Dutch Diabetes Federation as a supportive tool for managing recurrent hypoglycaemia.

### Trial status

This study is in the process of data collection. We expect the first trial results in the fall of 2015.

## Abbreviations

BGAT: Blood Glucose Awareness Training
EQ-5D: Euroqol 5 Dimensions
HbA1c: The standard measure glycaemic control, measuring glycaemic control over the last six to eight weeks
ICER: Incremental Cost-Effectiveness Ratio
QALYs: Quality-Adjusted Life-Years
RCT: Randomised Controlled Trail
T1DM: Type 1 diabetes mellitus
T2DM: Type 2 diabetes mellitus
